# Exploring perceptual differences and constraints in esports among American university students

**DOI:** 10.3389/fspor.2025.1669960

**Published:** 2025-11-14

**Authors:** Xianhua Luo, Li Chen, Juan Chang, Yong Yang, Seoyeon Lee

**Affiliations:** 1 Alfred Lerner College of Business & Economics, University of Delaware, Newark, DE, United States; 2 Department of Sport Management, Delaware State University, Dover, DE, United States; 3 Department of Physical Education, Hunan Normal University, Changsha, Hunan, China; 4 Department of Physical Education, Changsha University, Changsha, Hunan, China; 5 Department of Education, Kyungsung University, Busan, Republic of Korea

**Keywords:** collegiate esports, perceptual differences, constraints, factors, American university students

## Abstract

Esports have become a popular competitive gaming and intramural activity in higher education institutions. This study explores perceptual differences and constraints in Esports among American university students. 590 university students completed the Profile of Esports Perception—pre-validated questionnaire of five perceptual factors, namely attraction, economics, recognition, socialisation, and technicity. Multivariate analysis of variance and analysis of variance were utilised to assess the significant perceptual differences in Esports. Results revealed that recognition and socialisation were the lowest and highest scoring factors among the five perceptual factors, respectively. Findings indicated significant differences between male and female students, as well as sports players and non-players across attraction, economics, socialisation, recognition, and technicity. Significant differences were also observed in subgroups gender, playing status, and years played with respect to the negative factor of constraints. The findings may prove valuable to administrators of higher education institutions in understanding the opportunities and challenges of Esports.

## Introduction

Electronic sports (Esports) have gradually evolved from a niche hobby into a professionally formalized component of recreational and intramural activity in American post-secondary higher education. The National Association of Collegiate Esports (1) reports that over 240 higher education institutions in the US have their own Esports teams, include more than 5,000 collegiate athletes, and offer an estimated $16 million in Esports scholarships and aid. While this institutional legitimacy is well-documented, the social dynamics within collegiate Esports remain less understood. Existing research has effectively utilized theoretical frameworks and quantitative methods to establish findings on perceptual differences in gender, nationality, and culture among US college students engaged in Esports [e.g., (2–9)]. However, these studies have less frequently addressed the underlying perceptual and contextual constraints that shape student engagement with Esports within American universities. Therefore, there remains a need for a deeper extending the Chen et al. (10) framework by integrating a constraints dimension.

## Literature review

### Constraints of collegiate esports

Esports, traditionally a solitary and sedentary activity, conflict with conventional views of physical education and academic pursuits. Overcoming these stereotypes was pivotal in legitimising Esports as a valuable educational tool (11). The perceptions align directly with “intrapersonal constraints” as defined by leisure constraints theory (12), which are the psychological barriers individuals internalize about an activity. Absence of proper infrastructure is another barrier to the incorporation of Esports into educational institutions. Esports programs require dedicated spaces for practice and competition, high-quality gaming equipment, robust network infrastructure, and subject matter experts (13, 14). Unfortunately, formal training programs for Esports educators are lacking, hindering their abilities to harness the full educational potential of Esports (15, 16). Furthermore, the potential negative impact of increased screen time associated with Esports is a prevalent concern among educators and parents. Concerns regarding the effects of Esports on physical and mental health pose a significant barrier to its widespread adoption (17).

Historically, Esports have been male-dominated in both professional and educational settings (18), highlighting the need for inclusivity and diversity initiatives to address gender-related barriers (19). Moreover, unlike traditional sports, which often lead to scholarship and academic prestige, Esports have struggled to gain comparable acknowledgment. Establishing a framework that recognises and rewards accomplishments in Esports within the academic sphere is essential for fostering acceptance in educational institutions (20).

## Perception of esports

Esports have evolved from a recreational activity to a rapidly growing industry and have become increasingly embedded in higher education institutions. Therefore, Esports conceptualisations need reviewing. There has been widespread discussion on the definition of sports and how to distinguish sports activities from non-sports activities (21). According to Suits (22), sports must meet four essential criteria: physical exertion, physical skills, stability, and popularity. Hallmann and Griel (23) reported that social impact and competition outcomes are crucial characteristics. However, Esports are considered a machine-controlled game owing to the lack of full-body movement, organised competition management systems, and standardised rules for balanced and stable development (24). As a modern sport, it might not meet all the essential criteria of traditional sports; hence, further research is required on the perception of Esports among college students (25).

Gamer perceptions may be better understood by reviewing the literature on perception and analysing physiological sensations related to sports consumption. In addition, gender differences in perceptions in sports could be investigated by examining the awareness experiences of Esports players. Men have traditionally dominated the video game culture, while women were deemed to possess less skill, ambition, desire, and capability in sports (26). According to Jenny et al. (27), Esports are organised as competitive online games based on rules, skills, and a broad following; however, they lack physicality and institutionalisation. Esports have provided an interaction site for exploring human and non-human sporting performances (28). Jonasson and Thiborg (29) classified sports that use electronic devices as playing tools for organised competitive games. Esports have a more formational structure and popularity qualifications than sports (18). Lanier (30) noted that Esports were gradually being recognised as a worldwide competitive phenomenon.

**Figure 1:**
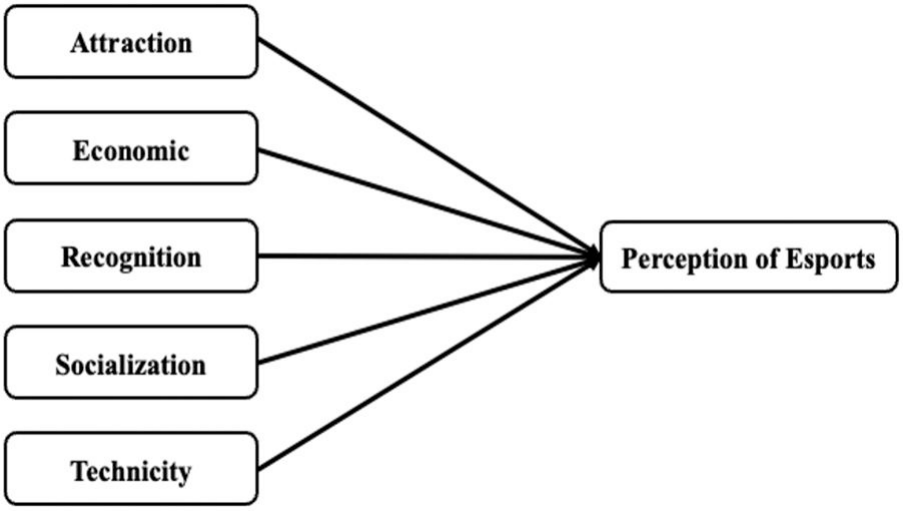
An overview of the esports perception scale for university students (10).

Considering current perception theories as a foundational basis, further research is required to understand the significant factors that influence perceptions of Esports. The current perception theory and conceptualisation of sports have established the significance of sports perception scales. Chen et al. (10) developed such a scale for college students that comprises five factors affecting their perception: attraction, economics, recognition, socialisation, and technicity (Figure 1).

The classical economic theory posits that economics plays a pivotal role in a capitalistic and free-market system wherein individuals are empowered to pursue their self-interest (31). Esports are profoundly influenced by financial considerations and economic valuations within global commerce (32). The industry is a lucrative and thriving sector, with its economic footprint expanding via meticulously organised competitions and extensive media coverage (33) owing to its fervent global fan base (34). Its dynamic commercial evolution hinges on the adept management of competitive events and promotional strategies (35). Esports have significantly bolstered financial capital within the electronics arts sector, with economic activities manifested through the establishment of sponsorship programs and strategic partnerships (33). Moreover, they have boosted investments in broadcasting and promotional endeavours (31) and heightened awareness of licensed merchandise and software copyright (36). Financial ventures have entered entertainment venues for competitive events, marking another facet of their economic influence (37).

The theory of recognition (38) proposes that an individual's perception of specific objects could be understood as an interplay between the mind and the body, influenced by contingent facts and the individual's brain state. This encompasses collective perceptions grounded in Esports’ factual and characteristic aspects and shared elements with traditional sports (23). “Play” was the fundamental essence and concept of electronic games (22; Tayler, 2012). Moreover, “institutionalisation” reflects the acceptance within media and governance spheres, establishment of managerial frameworks, development of governing bodies and regulations, formalisation of competitive structures, and facilitation of learning and coaching practices (11). Esports have been characterised by minimal physical exertion, controlling virtual avatars, and maintaining cognitive balance (39). When categorising activities based on a spectrum of physical-to-mental skill requirements, sports, such as boxing, require higher physical prowess with lower cognitive demands; conversely, other sports, such as shooting, demand higher mental acumen with less physical exertion. Both skillsets are essential for an activity to be classified as a sport (36).

Although skills and strategies in sports are crucial for individuals and teams to strive for victory (40), requisite equipment and facilities are indispensable to ensure the functionality of digital games (28). The socialisation theory posits that individuals require essential skills and knowledge to effectively integrate into communities and societies, which form a standard part of the group processes of living and life experiences (41). Esports could serve as a societal platform where members could actively participate, share interests and emotions, and engage in stress-relief, relaxation, and leisure activities (42, 43). Furthermore, Esports have the potential to influence individual's cognitive functions and human relationships through major social events, such as the Olympics or world championships, and foster interactions between the participants and audience (30, 42). Rise of social media is further intertwined with sports, amplifying their manifold social and cultural effects (43). Esports' evolving nature has spurred social diversity, shaped by their diverse fan base (32), and has been influenced by various social contexts and cultural heritages (40). It facilitates social functions and human interactions by hosting social events and strengthening relationships with the media through effective engagement (39).

Technicity pertains to the nature and quality of possessing technical skills and technology within a specific group (44). This characteristic distinguishes Esports from traditional sports (39). It encompasses elements of the Worldwide Web, online game functions, and broadband development, including standardisation, complex technicity infrastructure, and telecom engineering (45). It plays a crucial role as a supportive mechanism for gamers in Esports (39), provides transformative power, and bridges physical and social organs (46). Technical skills combined with human experience and behaviour in digital games enhance operational effectiveness and ensure skilful play and positive mental outcomes (46). Cyber athletes must demonstrate proficiency in hand-eye coordination, quick reactions, and equipment operations (45). Technical organs facilitate bodily and social functions in Esports, and technicity is a connecting force between the physical and social realms (46).

## Purpose

This study explores the perceptual differences and constraints toward Esports among American university students. Specifically, it addresses three research questions:

- What are the perceptions in Esports across important factors of attraction, economics, recognition, socialisation, and technicity among US university students?
- Would perceptions of Esports differ across gender, age, playing status, experience levels, and cost of playing?
- What are the perceptual differences in Esports constraints in US university campuses?

## Methods

This study utilised a quantitative research approach (47) to achieve a comprehensive view of the potential perceptual differences and constraints in Esports.

## Participants and instrument

Participants (*N* = 590), at full-time university students, were selected from a convenience sample of students studying at the private university of the University of Delaware (UD; student population >20,000) and the public university of Delaware State University (DSU; student population >7,000) along the US eastern coast. They were descriptively categorised into six groups: gender [men (*n* = 276, 46.8%), women (*n* = 314, 53.2%)], age [under 18 years (*n* = 25, 4.2%), 18–24 years (*n* = 340, 57.6%), and 25 years and older (*n* = 225, 38.1%)], ethnicity [Caucasian (*n* = 180, 30.5%), Asian (*n* = 248, 42%), African American (*n* = 145, 24.65%), other (*n* = 17, 2.9%)], education [2-year college (*n* = 189, 32%), 4-year college (*n* = 277, 46.9%), graduate study (*n* = 124, 21%)], cost [no cost (*n* = 324, 54.9%), some cost (*n* = 266, 45.1%), and play status [non-player (*n* = 352, 59.7%), player (*n* = 238, 40.3%; Table 1).

**Table 1:** Frequencies for independent variables (*N* = 590).

Group	Subgroup	Number of Participants (n)	Percentage (%)
Gender	Men	276	46.8
	Women	314	53.2
Age	Under 18	25	4.2
	18–24	340	57.6
	25 and above	225	38.1
Ethnicity	Caucasian	180	30.5
	Asian	248	42.0
	African American	145	24.6
	Other	17	2.9
Education	2 Years College	189	32.0
	4 Years College	277	46.9
	Graduate Study	124	21.0
Cost	No Cost	324	54.9
	Cost Some	266	45.1
Play status	Non-Player	352	59.7
	Player	238	40.3

The independent variable was gender (male or female) while the dependent variables were the scores for the five perceptual factors: attraction, economics, recognition, socialisation, and technicity. Additionally, the constraint factors were assessed separately. Participants were selected from 1,000 individuals, and 590 effective survey packages were obtained from the sample pool across higher education institutions. A structured questionnaire was distributed via university-affiliated email and in person at UD and DSU, with the questions grouped into the five perceptual factors.

This study used the Profile of Esports Perception [PEP; (10)]. It comprised 23 questions (items) categorised into the five perceptual factors with operational definitions: (a) “attraction” (questions 1–4) referred to the perception of Esports that could stimulate and attract students' interests and desires to engage in playing or entertaining Esports; (b) “economics” (questions 5–8) represented the perception of the economic impact and monetary gains via Esports promotions and competitive events; (c) “recognition” (questions 9–14) reflected the perception of Esports activities in a digital environment and recognition of their similar nature to sports, such as playfulness, equipment, institutionalisation and rules, strategy and outcome of sports competitions, and required physical skills; (d) “socialisation” (questions 15–19) was defined as the perception of Esports as having functions of social interaction, human relations, communication for social experience, and heritage of culture; and (e) “technicity” (questions 20–23) referred to the perception of sports competency in the required knowledge and skills of technical applications. Additionally, “constraints” (questions 24–31) were assessed and referred to the limitations and flaws inherent in Esports and the imperfections of electronic games. Each question (item) was rated on a 7-point Likert scale that ranged from 1 (*least agreed*) to 7 (*most agreed*).

## Procedure and analyses

The PEP had sound structural validity, as examined via a confirmatory factory analysis with satisfactory model fit indices: *X^2^/df* = 3.00, root mean square error of approximation = .065, goodness-of-fit index = .961, adjusted goodness-of-fit index = .961, comparative fit index = .945, non-normed fit index = .940 (10). Cronbach's alpha was used to assess its reliability or internal consistency and ranged from.806–.890 for the five subscales. Composite reliability estimates were ATT = .85, ECO = .72, REC = .87, SOC = .72, and TEC = .70, with an average of.78, which exceeded the satisfactory requirement (10). The survey package included an introduction paragraph, a demographic information sheet, and the PEP. For an English version of the PEP, three sports science and bilingual faculty members participated in the double-check correction and verification process, with an accuracy rate of >80%.

This study was consistent with the requirements of research ethics guided by the American Psychology Association. Furthermore, the instruments and procedures were reviewed and approved by the relevant institutional review board. A convenience sampling method was used to generate a relatively even number of participants. An electronic version of the survey was administered via the computer software Survey Monkey. The researcher distributed the survey package link through email. In total, 820 survey packages were returned (return rate, 82%). All returned surveys were verified, and surveys with missing data were excluded. Finally, 590 completed packages were analysed. Data were saved in an SPSS version 29 data file.

SPSS version 29 was used for statistical analyses, which included descriptive statistics, multivariate analysis of variance (MANOVA), and analysis of variance [ANOVA; (48)]. All assumptions of using MANOVAs were carefully checked and were satisfactorily valid (49). ANOVAs were conducted to test the effect of each independent variable on the five dependent variables.

## Results

**Figure 2:**
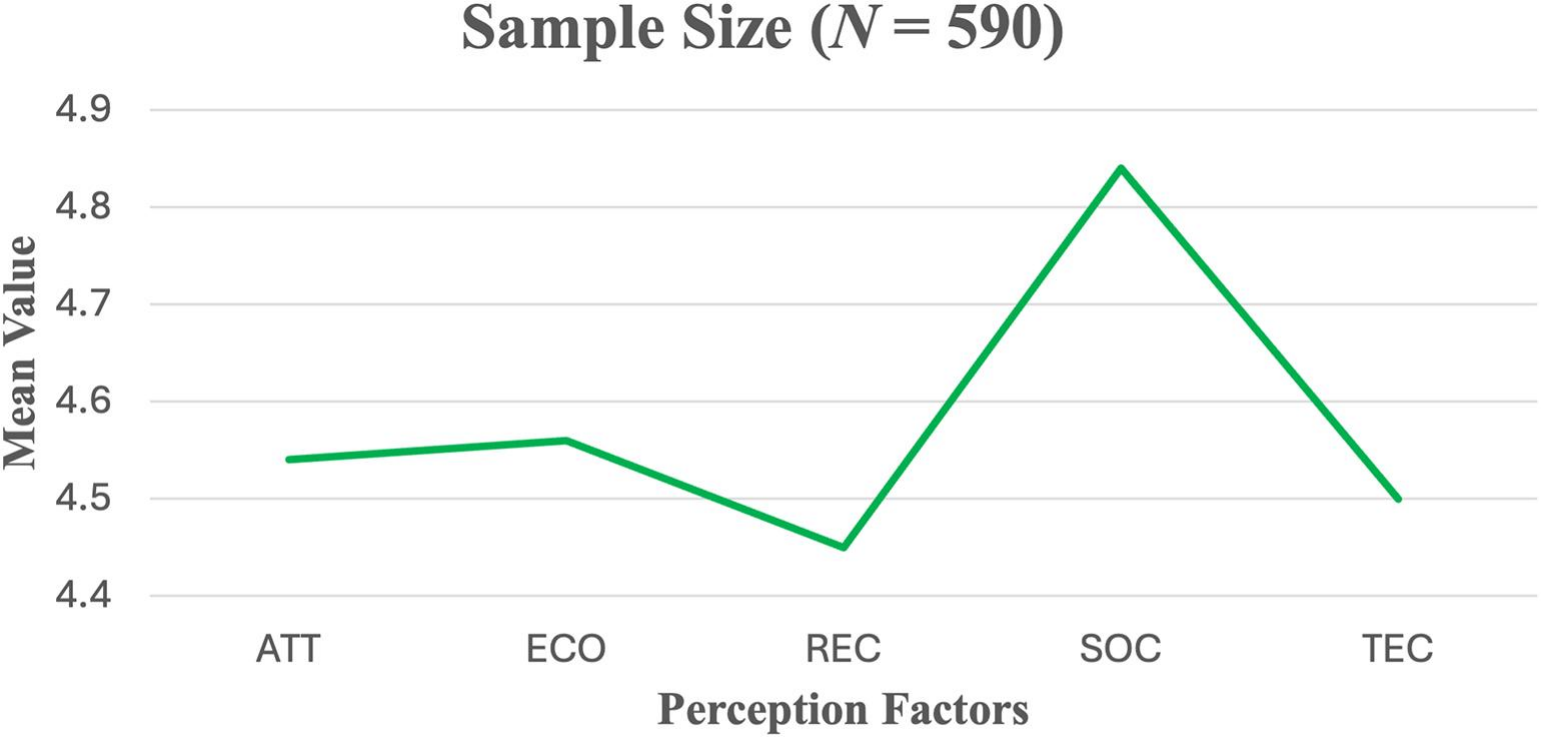
Mean scores of five factors of esports perception for all participants (*N* = 590).

Mean PEP rating scores for the entire dataset were highest for socialisation (4.97), followed by technicity (4.65), economics (4.64), recognition (4.54), and attraction (4.50; Figure 2).

Table 2 presents the means and standard deviations (SDs) of the perceptual factors of Esports based on a scale with 23 items. Socialisation had the highest mean rating (*M* = 4.84, *SD* = 1.36), followed by economics (*M* = 4.56, *SD* = 1.36), attraction (*M* = 4.54, *SD* = 1.49), and technicity (*M* = 4.50, *SD* = 1.26). Recognition had the lowest mean score (*M* = 4.45, *SD* = 1.54; Table 2).

**Table 2:** Means, standard deviations, and constraints of factors and items (*N* = 590).

Factors	Items	*M*	*SD*
**Attraction (ATT)**		**4**.**54**	**1**.**49**
ATT-1	The designs and contents of the Esports games are appealing	4.58	1.74
ATT-2	One has a sense of achievement when playing Esports games	4.68	1.71
ATT-3	Esports games are more immersive and challenging	4.52	1.61
ATT-4	Playing Esports can satisfy my curiosity	4.39	1.78
**Economics (ECO)**		**4**.**56**	**1**.**36**
ECO-5	Esports benefit the entire sports world economically	4.13	1.57
ECO-6	Esports games contribute to the electronic industry financially	4.66	1.63
ECO-7	Playing Esports can become a profitable profession	4.66	1.69
ECO-8	Esports have evolved into a complete business of the sports industry	4.79	1.72
**Recognition (REC)**		**4**.**45**	**1**.**54**
REC-9	Esports are similar to other sports because of their playfulness	4.61	1.76
REC-10	Esports are similar to sports because players use electronic equipment when playing Esports games	4.20	1.71
REC-11	Esports are similar to sports because strategies are applied when gaming	4.69	1.76
REC-12	Esports are similar to sports because they reveal outcomes (win/loss)	4.53	1.82
REC-13	Esports are similar to sports because they have established competition rules	4.59	1.83
REC-14	Esports are similar to sports because they require specific physical skills	4.08	1.77
**Socialisation (SOC)**		**4**.**84**	**1**.**36**
SOC-15	Playing Esports can foster the spirit of teamwork	4.97	1.67
SOC-16	The players can practice their ability to think, react, and coordinate	4.99	1.62
SOC-17	Playing Esports games can help me deal with distress	4.90	1.57
SOC-18	Playing Esports can help me extend my network and circle of friends	4.78	1.63
SOC-19	Esports has become part of our lives and culture	4.57	1.66
**Technicity (TEC)**		**4**.**50**	**1**.**26**
TEC-20	It is not hard for new players to master playing Esports games	4.18	1.67
TEC-21	E-sports players need systematic training to reach a certain competitive level	4.45	1.61
TEC-22	An excellent digital knowledge aids playing Esports	4.62	1.55
TEC-23	A certain level of intelligence is helpful for Esports to be competitive	4.75	1.63
**Constraints (CON)**		**4**.**30**	**1**.**25**
CON-1	Playing Esports can damage my communication with my family members or friends	4.22	1.88
CON-2	Playing Esports may waste my time or affect my normal life	4.44	1.93
CON-3	Being involved in Esports games can cause harm to my health	4.63	2.00
CON-4	The players use Esports games to gamble for money	3.97	1.80
CON-5	Young people are easily addicted to Esports games	5.85	1.78
CON-6	There is a lack of professional team management and coaches in Esports leagues	4.17	1.65
CON-7	There is not enough media attention given to Esports	4.09	1.69
CON-8	The Esports events are limited and lack support	4.08	1.60

Bold values indicate the mean (*M*) and standard deviation (*SD*) for each factor (Attraction, Economics, Recognition, Socialisation, Technicity, and Constraints), calculated by averaging the means of the individual items that belong to that factor.

Table 3 presents the means and SDs for subgroups gender, years, and cost. The scores of perceptions in Esports for most participants were positively skewed in high agreement on the importance of these factors. MANOVAs were used as the inter-correlation of perception factors measured the same variable (gender or years played). Subsequently, MANOVAs were conducted separately for each independent variable.

**Table 3:** Values of MANOVAs for variables gender and play status and ANOVAs for independent variables’ effect on constraints (*N* = 590).

MANOVA	Wilks’ lambda		*F*		*p*
Gender	.929		8.947		<.001
ANOVA	Sum of squares	*df*	Mean square	*F*	*p*
ATT	64.262	1	64.262	30.279	<.01
ECO	63.830	1	63.830	36.593	<.01
REC	80.310	1	80.310	35.871	<.01
SOC	47.337	1	47.337	26.513	<.01
TEC	33.949	1	33.949	22.092	<.01
MANOVA	Wilks’ lambda		*F*		*p*
Play status	.913		11.125		<.001
ANOVA	Sum of squares	*df*	Mean square	*F*	*p*
ATT	100.516	1	100.516	48.778	<.01
ECO	51.166	1	51.166	28.976	<.01
REC	78.389	1	78.389	34.962	<.01
SOC	60.333	1	60.333	34.216	<.01
TEC	43.510	1	43.510	28.615	<.01
ANOVAs of constraints	Sum of squares	*df*	Mean square	*F*	*p*
Gender	11.078	1	11.078	7.171	<.01
Play status	11.561	1	11.561	7.488	<.01
Years played	13.688	3	4.563	2.952	<.05

The independent variables age, ethnicity, cost, and education across the five perceptual factors were not significant (*p* > .05). However, significant differences were observed for gender and play status. The first set of MANOVAs revealed substantial differences for gender (Wilks' Lambda = .929, *F* = 8.947, *p* < .001). Follow-up univariate *F* tests were separately judged. They supported the findings with significant (*p* < .05) estimates for all the factors, with F values that ranged from 22.092 to 36.593 (*p* < .01). Male participants scored significantly higher on all the five factors than their female counterparts (Tables 3, 4).

**Table 4:** Means and standard deviations for subgroups of participants (*N* = 590).

Subgroups	ATT	ECO	REC	SOC	TEC	(CON)
Gender						
men (*n* = 276)	4.89 (1.39)	4.91 (1.24)	4.84 (1.42)	5.14 (1.22)	4.76 (1.25)	4.45 (1.11)
Women (*n* = 314)	4.23 (1.52)	4.25 (1.39)	4.10 (1.56)	4.58 (1.43)	4.27 (1.24)	4.18 (1.35)
Play Status						
Non-Player (*n* = 352)	4.20 (1.53)	4.31 (1.43)	4.15 (1.59)	4.58 (1.44)	4.28 (1.30)	4.19 (1.30)
Player (*n* = 238)	5.04 (1.29)	4.91 (1.16)	4.89 (1.35)	5.23 (1.13)	4.83 (1.13)	4.47 (1.60)
Years played						
0-Year (*n* = 275)	3.95 (1.48)	4.17 (1.39)	4.01 (1.53)	4.39 (1.43)	4.17 (1.23)	4.15 (1.31)
1-Year (*n* = 102)	4.87 (1.41)	4.75 (1.24)	4.71 (1.43)	5.07 (1.13)	4.72 (1.21)	4.55 (1.25)
2–4 Years (*n* = 124)	5.05 (1.29)	4.89 (1.28)	4.83 (1.50)	5.24 (1.23)	4.82 (1.29)	4.39 (1.19)
5-years & above (*n* = 89)	5.27 (1.16)	5.07 (1.19)	4.99(1.37)	5.40(1.15)	4.83(1.15)	4.36(1.10)

ATT, attraction; ECO, economic; REC, recognition; SOC, socialization; TEC, technicity; (CON), constraints.

The second set of MANOVAs revealed significant differences for the play status groups (Wilks' lambda = .913, *F* = 11.125, *p* < .001) across the five perceptual factors. Follow-up univariate *F* tests were also interpreted separately and supported these findings. Results revealed significant estimates for the five factors, with F values ranging from 28.615 to 48.778 (*p* < .001). Players scored significantly higher on all factors than non-players (*Ms* = 4.00 vs. 5.20 for ATT; 4.20 vs. 4.99 for ECO; 4.06 vs. 4.92 for REC; 4.53 vs. 5.22 for SOC; 4.27 vs. 4.78 for TEC; Tables 3, 4).

**Figure 3:**
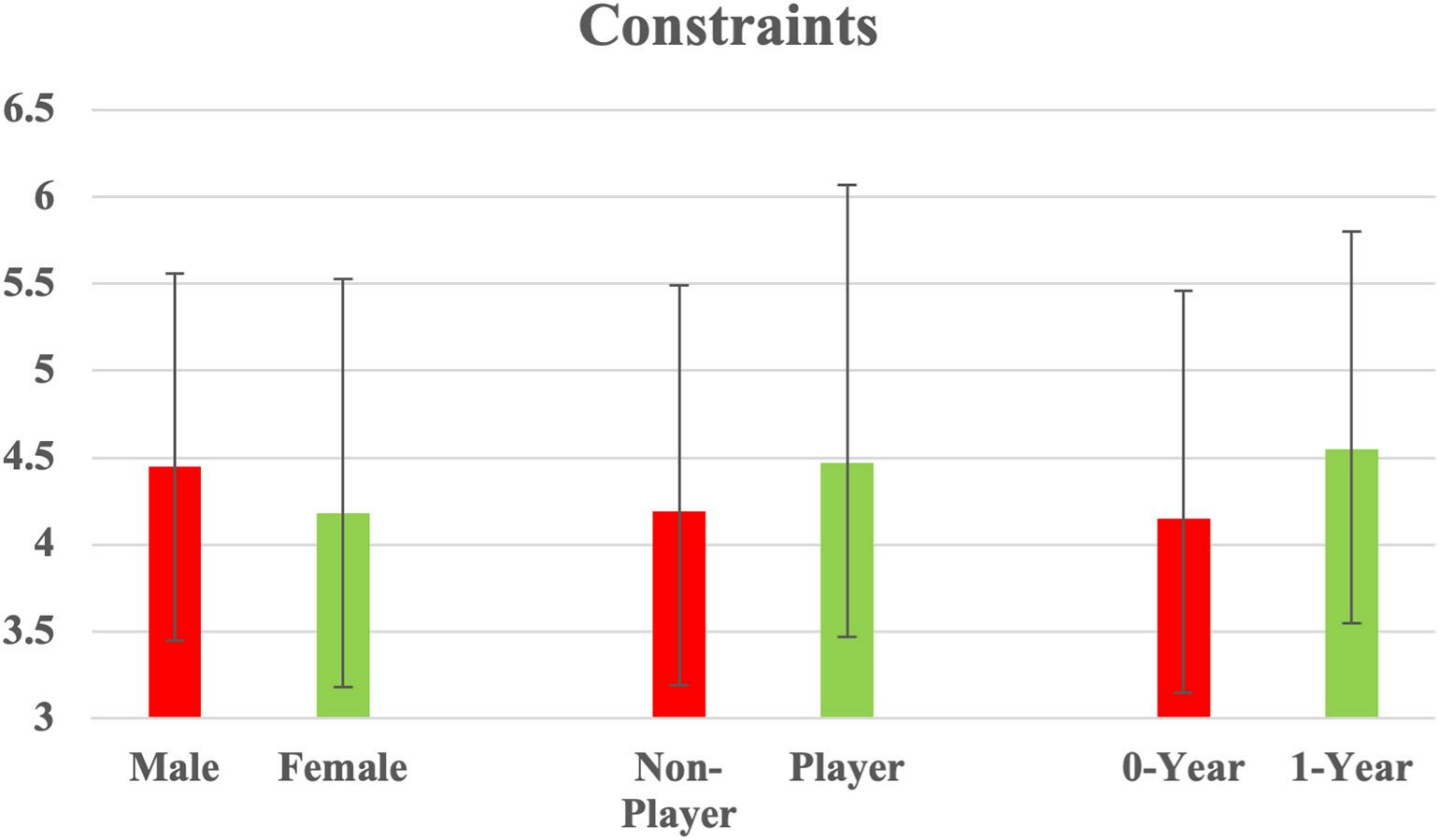
Significant differences in gender, play status, and years played with respect to constraints (*N* = 590).

Additionally, since the constraint factor was independent and in a non-multivariate situation [SPSS; (49)], ANOVAs were utilised to assess the differences. The independent variables age, education, and cost did not demonstrate significant differences across the dependent variable of constraints. However, the ANOVAs revealed substantial (*p* < .05) differences for the independent variables gender, play status, and years played, with F values that ranged from 7.171 to 7.488 (*p* < .01; Tables 3, 4). Detailed findings for these three independent variables are presented below (Figure 3):

- Gender: ANOVA revealed a significant difference in gender for constraints, with an F value of 7.171 (*p* < .01). Male participants scored significantly higher on the constraint factor than their female counterparts.
- Play status: ANOVA revealed a significant difference in play status for constraints, with an F value of 7.488 (*p* < .01). Players rated the constraint factor significantly higher than non-players.
- Years played: ANOVA revealed a significant difference in years played, with an F value of 2.952 (*p* < .05). A *post-hoc* Scheffé test was conducted to determine detailed difference and indicated that the 0-years subgroup scored significantly higher than the 1-year subgroup (*p* < .05). Furthermore, no significant difference was observed between comparisons in the other years played subgroups (*p* > .05; Tables 3, 4).

This study investigated the perceptions of Esports among university students in American higher education institutions. A quantitative survey was conducted with 590 participants to gather insights into their perceptions, constraints, and potential factors that affected their perception of Esports. These findings suggest a generally positive perception. Socialisation and recognition were the highest and lowest rated factors, respectively. Male participants scored significantly higher on all five factors of perception in Esports (attraction, economics, recognition, socialisation, and technicity) than their female counterparts.

Furthermore, non-players rated significantly lower than the Esports players on all five factors of perception. Constraints was scored significantly different by gender groups; males scored higher on constraints in Esports than females. Additionally, non-players scored significantly higher for constraints than the players. The 0-years subgroup scored significantly higher on constraints than the 1-year subgroup. Therefore, the results of the perception differences among university students could be a sound reference for guiding initiatives for collegiate sports development.

## Discussion

This study assessed the differences in the five perception factors in Esports among American college students. Results revealed that socialisation was the highest-scoring factor among the participants, while recognition was the lowest-scoring factor. Socialisation is an essential perceptual factor in Esports, as demonstrated in online communities, global reach, shared interests, streaming and content creation, competitive nature, and events and tournaments part of collegiate students’ entertainment activities. This is consistent with previous research by Kim et al. (50), who indicated that the sociocultural function of collegiate sports requires a diverse social platform for college students. Montemagni et al. (51) considered Esports a dynamic and innovative form of entertainment that could cultivate college students' collective sense of honour and competitive spirit and achieve the core value of socialism.

The lowest rating for Esports recognition reflects an entrenched mindset in traditional sports cognition. Perhaps the divergence in individual perceptions and knowledge capital still reflects the differences between conventional sports and Esports. This finding is similar to that of Pizzo et al. (9), who indicated that sports spectators have existing perceptions of cognitive skill similarities and differences in integrating traditional sports and Esports. The lowest score was rated for item 14 in recognition (Esports is like a sport because it requires minimal physical skills; *M* = 4.08). This result aligns with those of previous studies that identified physical skills as the primary characteristic of sports that could be demonstrated through physical activities requiring specific attributes, such as strength, speed, endurance, flexibility, and coordination (52).

However, Esports are organised as a competitive activity that uses electronic devices and related game software and requires coordination among hands, eyes, and brain (53). Motor abilities (54) and movement behaviour (53) in Esports competitions involve the depletion of body functions. The rating score reflected university students' individual perceptions based on their cultural background, prior knowledge, personal experiences, and biases. These findings confirm those of previous research that revealed that the perception and physical characteristics of Esports require improvement to enhance their recognition among university students (55).

This study found significant perceptual differences factors across gender. Female students rated the perception factors lower than their male counterparts. Men might often be more attracted to gaming and Esports than women. Historically, male players have been over-represented in gaming culture compared with female players (56). Lack of female representation in professional competitions, gaming media, and gaming communities may discourage female participation in Esports. These results are consistent with those of Darvin et al. (57) and Jang et al. (58), who reported that the phenomenon was owing to Esports consumption perception, habits, and market influence, which promoted the design of specialised gaming software related to sports gameplay intention. Video games and Esports content were possibly marketed in males with themes and storylines that appealed to their interests than female interests. A previous study on gender differences also reported that games’ social, cultural, and psychological benefits were perceived differently (7, 59).

Moreover, the dependent variable, playing status, was significant in the perception of all five factors. Non-players scored significantly lower for all five perception factors than sports players. Perhaps players were generally perceived to perform better in attention and reaction speed than non-players owing to their playing experience. According to Rai and Yan (45) and Boot et al. (60), non-players underperformed more frequently than players in perceived attention and performance. Esports players might perceive themselves as having better technical skills and competencies than non-players. Poulus et al. (61) reported that sports players believed that they had better hand-eye coordination, reaction time, and strategic thinking than non-players. Furthermore, Esports players perceived themselves as having superior technical skills and competencies owing to their extensive training, practice, and dedication to mastering their games (62). Esports players may have more cumulative experience in their respective domains than non-players. Christou (63) found that Esports players' playing experience affected usability and appealed more to positive perceptions than non-players. Perhaps human experience might help refine skills, understand strategies, anticipate outcomes, and deal with pressure situations. Their depth of knowledge could lead them to perceive Esports as sports owing to the competitive nature of the games (10).

Furthermore, significant differences were observed in constraints, a negative aspect of Esports perception, across gender, playing status, and years played. Constraints in Esports across gender groups could be owing to cultural deviation and related to their educational training and cultural background. Traditional gender roles and stereotypes may discourage females and non-binary individuals from participating in competitions (57). Kruthika (64) indicated that stereotypes discouraged female participation in video games and had nothing to do with their physical abilities. Male-dominated networks and teams may exclude women from game training and professional development opportunities. As a modern electronic competition, Esports have exhibited a male-dominated environment and professional development. Consequently, they lack support and resources for female players (57).

An Esports player can be constrained by various factors such as skill development, financial limitations, infrastructure, and time commitment required. A previous study indicated that skill development and learning curve of Esports were significant barriers to entry into and participation in Esports (20). According to the 2019 Global Esports Market Report (65), infrastructure constraints are challenging for developing regions. The Esports Ecosystem Report (65) highlighted financial barriers as a significant challenge for professional gamers. Taylor et al. (66) reported time commitment as a substantial barrier for amateur sports players in competitive gaming. Additionally, Esports participants with fewer years of experience might be constrained by skill development and proficiency, interest and engagement, and cognitive and physical differences. Esports players with more experience might react more quickly and have better hand-eye coordination than those with fewer years of experience.

Chen et al. (10) revealed that playing Esports requires hand-eye coordination and quicker reaction abilities to operate electronic game equipment. Esports tend to be more popular among players with more experience than those with fewer years of experience. According to Newzoo's (67) video game report, most Esports enthusiasm was among a player's generation. Experienced Esports players might have cognitive abilities and physical dexterity advantages compared with those with fewer years of experience. Maillot et al. (68) indicated that improvement of physical and cognitive abilities is necessary for less-experienced players to participate in interactive physical activities and video game training. Another reason could be that male players may have access to more opportunities, resources, or networks, which in turn could make them feel less constrained in their actions or decisions. Moreover, laws and regulations concerning employment, property, and legal rights differ between genders and could influence the perception of constraints that women face compared with men (69). Social norms and institutions may also limit women's access to resources, opportunities, and networks (70).

However, interestingly, the subgroup of players rated the constraint factor significantly higher than non-players. Esports players may need to manage their time more effectively to balance practice, competition, and commitments than non-players. According to Hamari and Sjöblom (20), Esports players must effectively manage their time to juggle practice, competition, and other responsibilities. This is supported by Delello et al. (71), whose research on collegiate players highlighted the significant impact of Esports on health and wellness habits, underscoring the real-world constraints of high-level participation. Conversely, the strong social rewards of participation on offset these constraints. The emphasis on socialization within collegiate Esports is further validated by recent multi-national studies. For instance, Delello et al. (72) found that participation in university Esports programs provided students with significant rewards in terms of community building and peer connections, reinforcing its role as a vital social platform. Similarly, Teixeira et al. (73) observed that the gaming habits of European university students were heavily influenced by the social dynamics and sense of belonging fostered within Esports communities. Possibly, many Esports games require players to have more vital teamwork and communication skills than non-players. Strong teamwork and communication skills can help develop the ability to collaboratively navigate constraints (20). Other reasons for this finding could be more opportunities for gamers to socialise. Cole and Griffiths (74) reported that gamers engage in more social interactions in video games, which may provide more opportunities to build strong friendships and emotional relationships with other gamers. Consequently, their perception of constraints could be much lower than that of non-players. Esports could enable social connections and enhance enjoyment. Hence, recognition of gaming as a significant social activity can increase the perceived value of socialisation. Esports can serve as a social channel for connecting individuals worldwide in shared gaming spaces to foster a sense of closeness, belonging, and security, which may offset perception constraints (75).

Additionally, the subgroup with 0-years of experience scored significantly higher on constraints than the 1-year of experience subgroup. Higher scores in the 0-years subgroup might indicate a more favourable or optimistic perception of sports among students who have not yet engaged in Esports. Kim et al. (76) demonstrated that Esports play was positively associated with perceived sportsmanship, which could contribute to a more favourable perception among those who had not yet engaged in it. Lower scores in the 1-year subgroup may reflect a more critical or realistic view after participants had been involved in Esports for a year. Involvement in Esports for a longer time may have revealed the significant commitment required to succeed and potentially lead to more realistic appraisals of one's abilities and the Esports landscape (24, 77). Subgroups of years played also exhibited a significant difference in constraints. Since gamers have played for more years, they perceived positive factors higher than those with fewer playing years. Nonetheless, they scored lower on constraints, a negative perceptual factor in Esports. This makes sense as students with more years of experience playing video games might form stronger bonds within the gaming community.

## Conclusions

This study on perception differences in Esports among American university students underscored the multifaceted benefits and potential of competitive gaming in higher education environments. Findings revealed that Esports influenced perception factors (attraction, economics, recognition, socialisation, and technicity) and perceptual constraints, which cultivated a sense of gender and playing experience among university students. Incorporation of Esports was aligned with the evolving landscape of digital skills and prepared university students for a competitive job market. As a platform for skill development and social engagement, Esports can be a valuable tool in enhancing the overall learning experience of students in higher education in America.

This study advocates for the continued exploration and implementation of Esports perceptions. Furthermore, it emphasises Esports' positive impact on students' academic and personal growth. It also underscores the importance of adapting dynamic interests and educational advancements and shaping the modern learning environment. Perception differences in Esports among university students could have various implications by combining the benefits and potential challenges of Esports. Positive implications are reflected in the perception differences across multiple aspects, such as skills development, industry relevance, student engagement, branding, marketing, and networking. Challenges are embodied in perceptual differences across various aspects, such as stereotypes, infrastructure costs, academic balance, inclusivity, and regulatory concerns. Collegiate Esports face multiple limitations related to financial constraints, academic priorities, diversity and inclusivity, external partnerships, and its dynamic market nature. Furthermore, the generalizability of these findings may be limited by the specific demographic and cultural context of the American college students sampled. Future research should employ a multidimensional research approach and investigate factors that motivate multicultural students to successfully perceive Esports in the university landscape.

## Data Availability

The original contributions presented in the study are included in the article/Supplementary Material, further inquiries can be directed to the corresponding author.
